# Incidence of Atrial Fibrillation or Arrhythmias After Patent Foramen Ovale Closure

**DOI:** 10.1016/j.jscai.2023.101173

**Published:** 2023-11-30

**Authors:** Keeley S. Ravellette, Jeff Gornbein, Jonathan M. Tobis

**Affiliations:** a David Geffen School of Medicine, UCLA, Los Angeles, California; b Statistics Core, Department of Medicine, David Geffen School of Medicine, UCLA, Los Angeles, California; c Department of Medicine, Division of Cardiology, UCLA, Los Angeles, California

**Keywords:** adult congenital heart disease, atrial fibrillation, occlusion devices, patent foramen ovale

## Abstract

**Background:**

Patients with a patent foramen ovale (PFO) who undergo percutaneous PFO closure are at a greater risk of developing atrial fibrillation (AF) compared with patients whose PFOs are managed medically. Postclosure AF appears to be well tolerated if treated but may increase the risk for stroke. Postclosure AF is reported to occur in 3.7% to 7.4% of patients; however, incidence across devices remains uncertain. This study aims to evaluate the frequency of postclosure AF, atrial flutter, and arrhythmias in 6 PFO closure devices.

**Methods:**

Four hundred forty-five patients underwent percutaneous PFO closure with appropriate follow-up between 2001 and 2021. The procedure was performed using Abbott Amplatzer PFO, Amplatzer ASD, Amplatzer Cribriform, NMT CardioSEAL, Gore Helex, or Gore Cardioform devices. Incidence of AF, atrial flutter, and arrhythmias were assessed by electrocardiogram within 6 months from closure. Multivariate logistic regression evaluated potential predictors of postclosure AF or atrial flutter.

**Results:**

Postclosure AF or atrial flutter occurred in 30 patients (6.7%) within 6 months, and its incidence was significantly different across devices. Gore Cardioform had the greatest frequency of postclosure AF or atrial flutter events (16.8%) compared with other devices. The Gore Cardioform device, larger device sizes, and male sex were associated with greater risk of postclosure AF or atrial flutter.

**Conclusions:**

Postclosure AF or atrial flutter was more likely to occur in the Gore Cardioform device, in males, and in patients who underwent PFO closure with larger devices. Although it is more effective for complete closure, the Gore Cardioform device was shown to be an independent predictor of postclosure AF or atrial flutter.

## Highlights

• Atrial fibrillation and atrial flutter are common complications following percutaneous patent foramen ovale closure.
• Incidence of postclosure atrial fibrillation or atrial flutter is related to the closure device used.
• The Gore Cardioform device has the most frequent postclosure atrial fibrillation or atrial flutter events.
• Males and larger devices confer increased risk of postclosure atrial fibrillation or atrial flutter.
• The Gore Cardioform independently predicts postclosure atrial fibrillation or atrial flutter.

## Central Illustration

**Figure:**
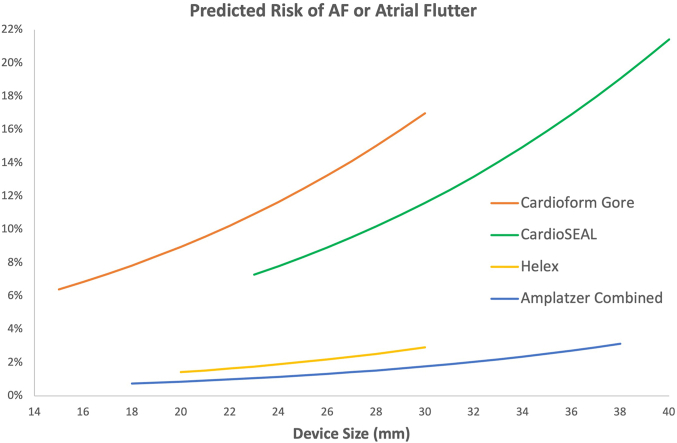

## Introduction

A patent foramen ovale (PFO) results from failure of the septum primum and septum secundum to fuse after birth. Anatomically significant PFO is the most common congenital cardiac lesion and affects up to 20% of the adult population.^1^ Several complications are associated with PFO, including migraine with aura,^2^ decompression illness,^3^ altitude sickness,^4^ platypnea-orthodexia syndrome,^5^ and most notably, paradoxical embolic stroke.^6^, ^7^, ^8^, ^9^, ^10^, ^11^ Patients with a PFO and history of ischemic stroke are encouraged to undergo PFO closure if they are under 60 years of age and there is no other identifiable cause of stroke. Randomized controlled trials have demonstrated that percutaneous transcatheter closure is a safe and effective option for PFO management and reduces risk of recurrent cryptogenic stroke compared with medical therapy alone.^12^, ^13^, ^14^

While percutaneous closure has become the standard for PFO-associated stroke management, it has also been shown to increase the risk of new-onset atrial fibrillation (AF). Chronic AF itself is a significant risk factor for stroke, and it also increases risk of other adverse cardiovascular events such as transient ischemic attack, myocardial infarction, and heart failure. The incidence of new-onset, postclosure AF most commonly occurs within the first 6 weeks and is reported in 3.7% to 7.4% of patients. A previous meta-analysis showed that AF is more common in patients with PFO who underwent percutaneous closure compared with those who were managed medically.^15^ The Reduction of Recurrent Stroke or Imaging-Confirmed TIA in Patients With Patent Foramen Ovale (REDUCE) clinical study investigated the incidence of postclosure AF in 2 PFO closure device types (Gore Cardioform and Helex) with various device sizes (30-35 mm vs 15-25 mm). The authors concluded that postclosure AF occurred more frequently in patients who underwent PFO closure with larger devices, but there were no associations between closure device type and AF incidence.^16^ A study from our group further investigated the incidence of AF in patients who underwent PFO closure with Gore Cardioform vs Helex and found the incidence of postclosure AF to be significantly higher in the Gore Cardioform group.^17^ Similarly, another study demonstrated that the Gore Cardioform device conferred an increased risk of postprocedural AF when compared with the Amplatzer PFO device.^18^

While previous studies have provided insight regarding incidence of AF in a subset of patients who underwent PFO closure with 2 devices, the incidence of AF, atrial flutter, and other arrhythmias compared across 6 widely used device types including Abbott Amplatzer ASD, Amplatzer PFO, Amplatzer Cribriform, NMT CardioSEAL, Gore Helex, and Gore Cardioform remains unknown. The aim of the current study was to evaluate the frequency of postclosure AF, atrial flutter, and arrhythmias across 6 PFO closure devices that were available for use in the United States. Clinical factors associated with AF and atrial flutter incidence were also explored.

## Methods

### Study design and patient population

Four hundred ninety-seven patients who underwent percutaneous closure of PFO at UCLA Medical Center between 2001 and 2021 were identified. Exclusion criteria consisted of a history of chronic AF or arrhythmias (n = 1). Patients who were lost to follow-up or lacked available records were also excluded (n = 51), resulting in a final sample of 445 patients. PFO closure was performed with the Abbott Amplatzer ASO, Amplatzer PFO, Amplatzer Cribriform, NMT CardioSEAL, Gore Helex, or Gore Cardioform devices sequentially as they became available over the time frame of the database review. The Helex and CardioSEAL devices are no longer manufactured but are included in the analysis to compare incidence of AF to devices currently utilized. Device sizes were obtained by measuring the edge-to-edge disc size. There were no incidences of procedural complications such as infection, pericardial effusion, or vascular access issues. Following closure, patients were prescribed dual antiplatelet therapy for 1 month followed by single-antiplatelet therapy for 1 year. This study was approved by the UCLA Institutional Review Board as an anonymous database review. Written informed consent was waived by the Institutional Review Board.

### Outcomes

The primary outcome of interest was incidence of AF and incidence of AF or atrial flutter within 6 months from PFO closure. All patients included in the study had close follow-up for at least 6 months following PFO closure. Detection of postprocedural arrhythmias was elucidated based on patient’s self-reported symptoms. Symptoms were further evaluated by electrocardiogram (ECG) or extended ECG monitoring, Apple Watch, or Kardia devices to elicit an ECG-based diagnosis. The duration of AF or atrial flutter was variable and unspecified given the episodes were unable to be assessed with continuous rhythm monitoring. However, most patients endorsed intermittent symptoms for minutes to hours. In the event of any persistent postprocedural AF or atrial flutter, even if for a brief duration, dual antiplatelet therapy was discontinued and patients were prescribed oral anticoagulation in addition to an antiarrhythmic. There were no patients that required electrical cardioversion. None of the patients with postclosure AF or atrial flutter had a recurrent stroke or other adverse event.

Secondary outcomes of interest were also explored, including incidence of premature atrial contractions (PAC), supraventricular tachycardia ([SVT] other than AF), and premature ventricular contractions (PVC). All medical records were reviewed for outcome classification and assignment of incident dates.

### Statistical analysis

#### Bivariate analysis

The *P* values for comparing continuous data between 2 groups were computed using *t* tests if the data followed the normal distribution. If the continuous variable did not follow the normal distribution the *P* value was computed using the Wilcoxon rank sum test. The *P* values for comparing categorical variables across groups were computed using Fisher exact test.

#### Multivariable analysis

The simultaneous association of device type and age with the AF or atrial flutter binary outcome was assessed using logistic regression. In addition to device type and age, the potential confounding effects of device size, gender, prior diagnosis of hypertension, diabetes hyperlipidemia, left ventricular ejection fraction <50%, left atrial volume index <35 mL/m^2^, left ventricular enlargement (defined as left ventricular diastolic volume >150 mL) and left ventricular diastolic dysfunction were simultaneously assessed using the Akaike information criterion (AIC). History of sleep apnea and pulmonary hypertension were also considered but were not included in the analysis since few subjects had these conditions. Thus, 11 potential predictors were simultaneously assessed. The model with the minimum AIC is reported. This is not exactly the same as the *P* < .05 or *P* < alpha significance criterion but is similar. Restricted cubic splines were used to determine if the effect of age or device size, the 2 continuous predictors, had a linear relation with the log odds of AF/atrial flutter.

Logistic model accuracy was assessed by computing the receiver operating characteristic curve area (concordance statistic = C) and the sensitivity and specificity at maximum accuracy where accuracy is defined as the average of sensitivity and specificity (accuracy = 0.50 sensitivity + 0.50 specificity). The 95% CIs for odds ratios (ORs) are reported.

## Results

### Baseline characteristics of the study population

Baseline demographic characteristics of the study cohort are presented in Table 1. Of the 445 patients, 50.6% were male, and mean age was 53 ± 14 years. Patients were more commonly referred for a cerebrovascular accident (73.7%), followed by transient ischemic attack (9.4%). The greatest proportion of closures was performed using the Helex device (32.1%), and the mean closure device size used was 26.3 ± 4.9 mm.

**Table 1:** Baseline characteristics of the study population.

Variable	N = 445
Age at procedure, y	53 ± 14
Male sex	225 (50.6%)
Hypertension	123 (27.6%)
Diabetes	39 (8.8%)
Hyperlipidemia	171 (38.4%)
Sleep apnea	4 (0.90%)
Pulmonary hypertension	8 (1.80%)
LA volume index >35 mL/m^2^	20 (4.5%)
LV diastolic volume >150 mL	13 (2.92%)
Left ventricular ejection fraction <50%	22 (4.94%)
LV diastolic dysfunction	29 (6.52%)
Reason for referral	
Cerebrovascular accident	328 (73.7%)
Transient ischemic attack	42 (9.4%)
Migraine headache	30 (6.7%)
Arterial desaturation	29 (6.5%)
Myocardial infarction	4 (0.9%)
Embolus, noncerebrovascular accident, or myocardial infarction	7 (1.6%)
Decompression illness	1 (0.2%)
Transient neurologic deficit	4 (0.9%)
Device type	
Amplatzer ASD	24 (5.4%)
Amplatzer Cribriform	19 (4.3%)
Amplatzer PFO	108 (24.3%)
CardioSEAL	26 (5.8%)
Helex	143 (32.1%)
Gore Cardioform	125 (28.1%)
Device size, mm	26.3 ± 4.9

Values are n (%) or mean ± SD.

LA, left atrium; LV, left ventricle.

Postclosure AF or atrial flutter occurred in 30 (6.7%) of all patients. The AF or atrial flutter group had a greater proportion of male patients compared with the non-AF or atrial flutter group (73.3% vs 48.9%; *P* = .01). In the AF or atrial flutter group, 70% of patients’ PFO were closed with a Gore Cardioform device, while in the non-AF or atrial flutter group, the Helex device was more commonly used (33.8%). There were no statistically significant differences in hypertension, diabetes, hyperlipidemia, left ventricular ejection fraction <50%, left atrial volume index <35 mL/m^2^, left ventricular enlargement, left ventricular diastolic dysfunction, or reason for referral (Table 2).

**Table 2:** Bivariate analysis of baseline characteristics of patients with postclosure AF or atrial flutter vs without postclosure AF or atrial flutter.

Variable	AF or atrial flutter (n = 30)	No AF or atrial flutter (n = 415)	Odds ratio	*P* value
Age at procedure, y	54 ± 13	53 ± 14	1.006^a^	.78
Male sex	22 (73.3%)	203 (48.9%)	2.87	.013
Hypertension	9 (30%)	114 (27.5%)	1.13	.83
Diabetes	2 (6.67%)	37 (8.92%)	0.73	.99
Hyperlipidemia	12 (40%)	159 (38.3%)	1.07	.85
Sleep apnea	0	4 (0.96%)	–	.99
Pulmonary hypertension	0	8 (1.93%)	–	.99
LA volume index >35 mL/m^2^	3 (10%)	17 (4.1%)	2.6	.14
LV diastolic volume >150 mL	0	13 (3.13%)	–	.99
LVEF <50%	1 (3.33%)	21 (5.06%)	0.65	.99
LV diastolic dysfunction	1 (3.33%)	28 (6.75%)	0.48	.71
Reason for referral				.31
Cerebrovascular accident	22 (73.3%)	306 (73.7%)	–	–
Transient ischemic attack	4 (13.3%)	38 (9.2%)	–	–
Migraine headache	1 (3.3%)	29 (7.0%)	–	–
Arterial desaturation	1 (3.3%)	28 (6.7%)	–	–
Myocardial infarction	0	4 (1.0%)	–	–
Embolus, noncerebrovascular accident or myocardial infarction	2 (6.7%)	5 (1.2%)	–	–
Decompression illness	0	1 (0.2%)	–	–
Transient neurologic deficit	0	4 (1.0%)	–	–
Device type				
Amplatzer ASD	0	24 (5.8%)	1-Reference	–
Amplatzer Cribriform	0	19 (4.6%)	1-Reference	–
Amplatzer PFO	3 (9.7%)	105 (25.4%)	1-Reference	–
Helex	3 (9.7%)	140 (33.8%)	1.06	.95
CardioSEAL	3 (9.7%)	23 (5.6%)	6.43	.03
Gore Cardioform	21 (70%)	104 (25%)	9.96	.003
Device size, mm	28.1 ± 4.13	26.9 ± 4.13	1.07	.08

Values are mean ± SD or n (%). All devices are compared to the combined Amplatzer devices for odds ratios.

AF, atrial fibrillation; LA, left atrium; LV, left ventricle; LVEF, left ventricular ejection fraction.

a Odds per 1 unit (y or mm) increase.

### Incidence of postclosure AF, atrial flutter, and arrhythmias across devices

At 6 months postclosure, there was a significant difference in incidence of AF across all 6 devices (*P* < .001; Table 3). The Gore Cardioform group had the greatest proportion of patients with AF (15.2%), followed by CardioSEAL (11.5%). The Amplatzer ASD and Amplatzer Cribriform groups had no occurrence of postclosure AF. Of the patients with postclosure AF, 1 had a prior history of palpitations. None of the patients who developed postprocedural AF had prior history of paroxysmal AF.

**Table 3:** Incidence of postclosure AF, atrial flutter, and arrhythmias across devices.

Outcome	N	Amplatzer ASD	Amplatzer Cribriform	Amplatzer PFO	CardioSEAL	Helex	Gore Cardioform	*P* value
AF	27	0	0	3 (2.8%)	3 (11.5%)	2 (1.4%)	19 (15.2%)	<.001
Atrial flutter	3	0	0	0	0	1 (0.7%)	2 (1.6%)	.78
AF or flutter	30	0	0	3 (2.8%)	3 (11.5%)	3 (2.1%)	21 (16.8%)	<.001
Palpitations	76	3 (12.5%)	5 (26.3%)	12 (11.1%)	7 (26.9%)	30 (21.0%)	19 (15.2%)	.15
PAC	5	0	0	4 (3.7%)	0	0	1 (0.8%)	.18
SVT	4	0	0	1 (0.9%)	0	0	3 (2.4%)	.48
PVC	6	0	0	0	0	5 (3.5%)	1 (0.8%)	.33

AF, atrial fibrillation; PAC, premature atrial contraction; PVC, premature ventricular contraction; SVT, supraventricular tachycardia other than AF.

There were no significant differences across the groups in terms of incidence of atrial flutter. Two patients in the Gore Cardioform group (1.6%) and 1 in the Helex group (0.7%) developed atrial flutter. One patient with atrial flutter in the Gore Cardioform group had a prior history of SVT. Looking at the combined outcome, the incidence of AF or atrial flutter was significantly different across the devices (*P* < .001). Gore Cardioform had the greatest proportion of patients with postclosure AF or atrial flutter (16.8%) followed by CardioSEAL (11.5%). There were no incidences of postclosure AF or atrial flutter in the Amplatzer ASD and Amplatzer Cribriform groups.

All device types had patients who endorsed palpitations (n = 76, 17.1%). Of these, 8 had a prior history of palpitations, 3 had prior AF, and 1 had a previous unspecified atrial arrhythmia. Four patients had SVT (0.9%), of which, 1 had a prior history of SVT. Five patients had PAC (1.1%), and 6 had PVC (1.3%). There were no significant differences in incidence of atrial flutter, PAC, SVT, or PVC between the groups. Three hundred twenty-three (72.6%) patients did not develop AF, atrial flutter, or arrhythmias. Three had prior paroxysmal AF, 2 had a history of atrial flutter, and 1 had prior atrial tachycardia.

### Predictors of postclosure AF or atrial flutter

The impact of each potential predictor on AF or atrial flutter incidence was first assessed 1 at a time (Table 2). All 3 Amplatzer devices were combined into 1 category, given there was a small sample size and no AF or atrial flutter events in patients with Amplatzer ASD or Amplatzer Cribriform devices, and only 3 events in Amplatzer PFO. The Helex device was associated with a slight, nonsignificant increased risk of AF or atrial flutter compared with the combined Amplatzer devices (OR, 1.06; *P* = .946), while the Gore Cardioform and CardioSEAL devices were associated with increased odds (OR, 9.96; *P* < .003 and OR, 6.43; *P* = .028). Furthermore, each 1 mm increase in device size was associated with 7% increased odds for AF or atrial flutter (OR, 1.07; *P* = .08). Regarding demographic and clinical characteristics, only male sex was associated with an increased odds of AF or atrial flutter (OR, 2.87; *P* = .013) ignoring all other factors.

A multivariate logistic model assessed the collective impact of up to 11 potential predictors on the incidence of AF or atrial flutter. The minimum AIC logistic model found that male sex, device type, and device size were simultaneously significant. Given device type, device size, and sex, the other potential predictors were not significant by AIC. The model results are shown in Table 4. Controlling for gender and device size, both Gore Cardioform and CardioSEAL were associated with increased odds of AF or atrial flutter when compared with the combined Amplatzer devices (OR, 11.36; 95% CI, 3.11-41.6; *P* = .0002 for Gore) and (OR, 7.29; 95% CI, 1.34-39.6; *P* = .021 for CardioSEAL). Helex also was associated with increased odds compared with Amplatzer (OR, 1.67; CI, 0.29-9.61 0; *P* = .57) but this was not statistically significant. Male vs female sex was associated with an increased odds of AF or atrial flutter (OR, 2.47; 95% CI, 1.04-5.91; *P* = .014). The odds of AF or atrial flutter increased as device size increased in all device types (Central Illustration). The OR = 1.08 per mm increase in device size (CI, 0.96-1.21; *P* = .23). While the device size *P* value was not less than .05, inclusion of device size lowered the AIC. Moreover, device size is a known risk factor, so it was retained in the model. The multivariate logistic model had a sensitivity of 79.3% and specificity of 70.6% for an overall accuracy of 75.0%. The model receiver operating characteristic curve area (concordance statistic) was C = 0.797.

**Table 4:** Multivariable logistic model demonstrating the impact of device type, device size, and male sex on atrial fibrillation or atrial flutter.

Variable	Odds ratio	95% CI	*P* value
Device			
Helex vs Amplatzer Combined	1.67	(0.29-9.61)	.57
Gore Cardioform vs Amplatzer Combined	11.36	(3.11-41.59)	.0002
CardioSEAL vs Amplatzer Combined	7.29	(1.34-39.56)	.02
Device size, per mm	1.08	(0.96-1.21)	.22
Male sex	2.47	(1.04-5.91)	.04

**Central Illustration:**
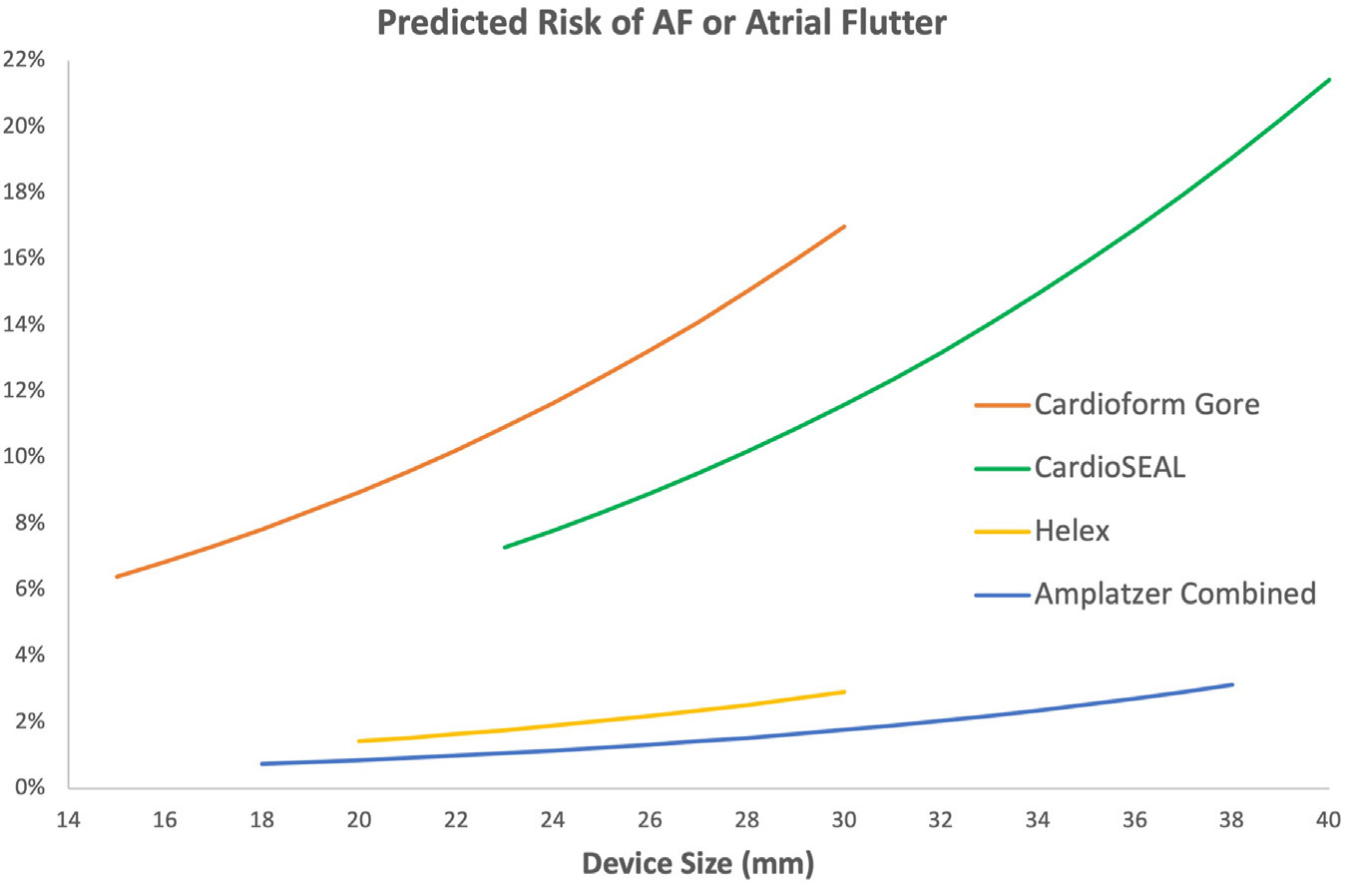
Multivariable logistic model for risk of atrial fibrillation (AF) or atrial flutter by device type and device size controlling for sex. The risk of AF or atrial flutter increased as device size increased in all device types. The effect of device size was allowed to vary across the 4 device types.

## Discussion

This study examined the frequency of AF, atrial flutter, or arrhythmias within 6 months after percutaneous PFO closure as well as the association between the type of closure device and potential predictors of atrial arrhythmias. Among 445 patients who underwent PFO closure between 2001and 2021, the overall incidence of postclosure AF was 6.1% and the incidence of postclosure AF or atrial flutter was 6.7%. This finding is similar to the reported postclosure AF incidence rate of 7.4% in the REDUCE trial but is higher than reported in the Randomized Evaluation of Recurrent Stroke Comparing PFO Closure to Established Current Standard of Care Treatment (RESPECT) trial (1.4%) and Closure of Patent Foramen Ovale or Anticoagulants Versus Antiplatelet Therapy to Prevent Stroke Recurrence (CLOSE) trial (4.6%). A recent systematic review and meta-analysis that included 6 randomized controlled trials and 26 observational studies reported that the incidence of postclosure AF was 3.7 patients per 100 patient-years of follow-up. The differences in reported incidences may be explained in part due to differences in methods of detection of arrhythmias. The true incidence of postprocedure AF and arrhythmias is likely to be more closely approximated in patients with continuous rhythm monitoring devices. Indeed, a recent study analyzing 225 patients with implantable or external loop recorder monitoring following PFO closure found the incidence of new-onset AF, atrial flutter, or SVT to be 20.9% within 28 days following the procedure.^19^

The mechanical and material characteristics of the devices also appear to play a role in the development of postprocedural AF. In the present study, the Gore Cardioform device had the highest incidence of postclosure AF or atrial flutter (16.8%), followed by CardioSEAL (11.5%), Amplatzer PFO (2.8%), and Helex (2.1%). This finding is consistent with the REDUCE trial and a previous study which showed a higher risk of postclosure AF in the Gore Cardioform device compared with Amplatzer or Helex.^16^^,^^17^^,^^20^ Furthermore, our study demonstrates that the Cardioform Gore device is an independent predictor of postclosure AF or atrial flutter when controlling for clinical, demographic, and device-specific characteristics. This finding may be explained, in part, by the unique structural properties of the Gore Cardioform device. The Gore Cardioform device is designed to generate an increased closing force which allows for better grip on the atrial septum and a decreased risk of device embolization or misalignment. These engineering changes of this device are associated with improved benefit of decreased residual shunting compared with the Amplatzer device (2% vs 15%).^17^ However, the force the device exerts on the atrial septum may produce more stress, irritation, and a local inflammatory response which may explain the increased incidence of AF and other arrhythmias. Furthermore, no studies have indicated an association between immediate postclosure AF and recurrent stroke. The AF that occurs in 2 to 4 weeks postclosure appears to be intermittent, transient, and dissipates by 10to 12 weeks consistent with the course of inflammation after device implantation.^19^^,^^20^ Currently, there are no clear treatment guidelines for patients with postclosure AF for the prevention of stroke. The patients in the current study were given anticoagulation and antiarrhythmic medication for 2 to 3 months, and there were no instances of recurrent stroke, suggesting that postclosure AF is transient with a relatively favorable prognosis.^21^ The clinical benefit of decreased residual shunting must be weighed against a greater risk of development of AF and arrhythmias when considering the use of the Gore Cardioform device.

Unexpectedly, age was not significantly different between patients with postclosure AF or atrial flutter compared with those without, and it was not a statistically significant predictor of postclosure AF or atrial flutter in both the bivariate and multivariate analysis. It has previously been shown that age is a strong predictor of AF, but that likely represents a connection with underlying heart disease or interstitial fibrosis of aging.^22^ The vast majority of patients with device-closed PFO are younger than 60 years. Yet, it is useful to note that people above the age of 40 years had no greater risk of developing AF compared with younger patients. This can be helpful when discussing the risks of the procedure with an individual patient.

Multivariate analysis demonstrated that device size was associated with increasing risk of AF or atrial flutter in all device types. The association between device size and risk of AF or atrial flutter may be explained in part, by device-induced atrial mechanical stretch leading to a local inflammatory response or electrical irritation that in turn increases susceptibility to arrhythmias.^23^^,^^24^ This is likely not the only contributing factor resulting in postclosure arrhythmias. Gaspardone et al^25^ analyzed the association between mechanical PFO closure using either Amplatzer PFO, StarFlex (NMT), Intrasept (Cardia Inc.), BioSTAR (NMT), or Occlutech (Occlutech International AB) devices and new-onset postclosure arrhythmias in a group of 221 patients. Patients underwent preclosure rhythm and postclosure rhythm monitoring using 24-hour or 48-hour Holter-ECG recording or external loop recorder monitoring. PFO closure was not found to be an inducer of 3 to 6 month postclosure atrial arrhythmias, and the only predictor of postclosure arrhythmias was presence of arrhythmias prior to closure.^25^ Krishnamurthy et al^20^ studied the incidence and time course of postclosure AF in a select group of high-risk patients who received an implantable loop recorder prior to PFO closure. Of the patients with an implantable loop recorder, 37% developed postclosure AF, the majority of which occurred within the first 4 weeks and resolved within 12 weeks after closure. Of the patients with implantable loop recorders, those with postclosure AF were older at the time of device implantation than those who did not develop postprocedure AF (mean age = 62 vs 52 years). Older age at the time of device implantation was associated with higher rates of postclosure AF. This finding along with the selected patient cohort are likely explanations for the high incidence of postclosure AF observed in the study.^20^

Previous studies have established that men are more susceptible to the development of AF compared with women; however, the cumulative lifetime risk is comparable given women have a greater life expectancy.^26^ Further, on average, women develop AF 10 years later than men, and men develop postoperative AF at higher rates than women.^26^^,^^27^ The REDUCE trial found male sex to be an independent predictor of postprocedural AF in patients who underwent closure with either Gore Cardioform or Helex devices (OR = 3.45, *P* < .01). Similarly, Guedeney et al^19^ found the incidence of AF, atrial flutter, and supraventricular tachycardias post-PFO closure to be more frequent in male patients compared with females.^19^ Our study aligns with the current literature and affirms the association between male sex and increased risk of AF or atrial flutter.

### Limitations

The first limitation of the study is that palpitations were self-reported by patients at follow-up visits and were then evaluated by ECG, extended ECG monitoring, Apple Watch, or Kardia devices to confirm a diagnosis. External and implantable loop recorders afford a more comprehensive assessment of postprocedural AF given they are more sensitive compared with detection based on patient-reported symptoms alone. The use of loop recorders as a means of detection of AF in stroke survivors was noted as a Class IIa recommendation in the 2016 European Society of Cardiology guidelines.^28^ Given that our study period began in 2001 prior to the recommended use of loop recorders, we were unable to use this as a means of detection of arrhythmias in the postprocedural period.

Furthermore, our study had a small number of AF and atrial flutter events, which yielded a low statistical power to identify potential predictors of AF and atrial flutter. This may be in part why age was not significant.

## Conclusion

The incidence of AF or atrial flutter within the first 6 months post-PFO closure in all patients was 6.7%. Patients who underwent PFO closure with the Cardioform Gore device had a higher frequency of postclosure AF or atrial flutter events compared with other devices (16.8%), and the Cardioform Gore device was found to be an independent predictor of postclosure AF or atrial flutter. The risk of postclosure AF or atrial flutter also appears to be greater in male patients and those who underwent PFO closure with larger devices. In this observational study, the presence of AF was transient and was not associated with any adverse events. The Gore Cardioform is still our preferred PFO closure device because of its reliable closure with minimal residual shunt, but the patients ought to be advised about the increased risk of developing transient AF.
